# Increasing effect of Tangzhiqing formula on IRS-1-dependent PI3K/AKT signaling in muscle

**DOI:** 10.1186/1472-6882-14-198

**Published:** 2014-06-21

**Authors:** Jing Gao, Jian Li, Yating An, Xuefeng Liu, Qian Qian, Yanlin Wu, Yi Zhang, Tao Wang

**Affiliations:** 1 Tianjin State Key Laboratory of Modern Chinese Medicine, 312 Anshanxi Road, Nankai District, Tianjin 300193, China; 2 Institute of Traditional Chinese Medicine, Tianjin University of Traditional Chinese Medicine, 312 Anshanxi Road, Nankai District, Tianjin 300193, China; 3 Key Laboratory of Pharmacology of Traditional Chinese Medical Formulae, Tianjin University of Traditional Chinese Medicine, Ministry of Education, 312 Anshanxi Road, Nankai District, Tianjin 300193, China

**Keywords:** IRS-1-dependent PI3K signaling pathway, Insulin resistance, *Paeonia lactiflora*, *Morus alba*, *Nelumbo nucifera*, *Salvia miltiorrhiza*, *Crataegus pinnatifida*

## Abstract

**Background:**

Tangzhiqing fomula (TZQ-F), the mixture of Red Paeony root, Mulberry leaf, Lotus leaf, Danshen root and Hawthorn leaf, regulates the abnormal glucose and lipids in prediabetic patients. In this study, we focus on the mechanism of TZQ-F and its fractions on glucose metabolism.

**Methods:**

After orally administration of TZQ-F for 4 weeks in KK-A^y^ mice, we dissected out the liver and muscle, and employed PCR and western blotting to screening the PI3K/AKT pathway. The following PI3K/AKT signaling pathway were performed in L-6 myotube and HepG2 cells.

**Results:**

In the liver of KK-A^y^ mice, no significance was observed on PI3K, AKT and their phosphorylation between TZQ-F and controls , while, in the muscle, up-regulation of PI3K, AKT, Glycogen synthase (GYS) and their phosphorylation type, as well as GluT4, was deteced in TZQ-F. In HepG2 cells, TZQ-F increased IRS-2 by 10 folds, without interrupting AKT, IRS-1 and GluT4. In L-6 myotube cells, TZQ-F and its fractions treatment significantly increased IRS-1 and AKT at mRNA level.

**Conclusion:**

TZQ-F prevents pre-diabetes through increasing effect on IRS-1-dependent PI3K/AKT signaling pathway in muscle.

## Background

After a meal, glucose stimulate glucose-uptaking in the liver and peripheral muscle tissues to oxidized glucose to carbon dioxide and water or converted to glycogen for energy storage. In the early stages of type 2 diabetes, significant decreasing of glycogen synthesis in muscle is the distinguishing characteristic for the insulin resistance [1]. Glycogen biosynthesis and consumption involves several signaling pathways, such as Adenosine 5’-monophosphate-activated protein kinase (AMPK) [2], Phosphoinositide 3-kinases (PI3K) [3], Wnt/β-catenin [4], Mitogen-activated protein kinase (MAPK) [5]. Further, AMPK and PI3K directly regulate glycogen synthase (GYS) to control glycogen metabolism and glucose storage.

PI3K is a phosphorylase converting inositol 4,5-bisphosphate to inositol 1,4,5-trisphosphate, which is a second messenger, playing a crucial role in transferring a chemical signal received by the cell, such as from a hormone, neurotransmitters, growth factors and hypertrophic stimuli [6]. PI3K signaling pathway consists of PI3K and its downstream proteins, including serine/threonine protein kinases (AKT), mammalian target of rapamycin (mTOR) and glycogen synthase kinase (GSK) et al., has been linked to an extraordinarily diverse group of cellular functions, such as cell growth, differentiation, survival and intracellular trafficking [7]. PI3K signaling pathway play important roles in a large number of human diseases, such as cancer [8], metabolic diseases [9] and inflammation [10].

Tangzhiqing formula (TZQ-F), a traditional Chinese medicine, contains five herbs, Red Paeony root, Mulberry leaf, Lotus leaf, Danshen root and Hawthorn leaf. In clinical, TZQ-F is widely used for prediabetes. Previously, we found that TZQ-F showed significantly reduced effects on serum glucose (GLU) and triglyceride (TG) levels in high- carbohydrate/ high- fat diet rats [11] and genetic type 2 diabetes model KK-A^y^ mice [12]. The effect was related to glucose and lipid absorption inhibition, and free radical scavenging. The effects on glucose and lipid homeostasis is mediated, at least in part, through AMPK signaling pathway.

As a further study, in this paper, we demonstrate that glucose metabolism regulation effect of TZQ-F mediated AKT activation on IRS-1-dependent PI3K signaling pathway in muscle.

## Methods

### Plant material and fractions preparation

As reported in the last literature [11], Red Paeony root, Mulberry leaf, Lotus leaf, Danshen root and Hawthorn leaf were purchased from Yonggang Chinese Medicine Co. Ltd. Bozhou, China, and identified by Dr. Tianxiang Li at Tianjin University of Traditional Chinese Medicine (TUTCM) as *Paeonia lactiflora* Pall., *Morus alba* L., *Nelumbo nucifera* Gaertn., *Salvia miltiorrhiza* Bunge., and *Crataegus pinnatifida* Bge., respectively. All the voucher specimens were deposited at the Institute of Traditional Chinese Medicine of Tianjin University of Traditional Chinese Medicine.

Preparation and quality control of fractions were described as our previous report [11], which were Red Paeony total saponins fraction, Lotus leaf total alkaloids fraction, Lotus leaf total flavonoids fraction, Mulberry leaf total alkaloids fraction, Mulberry leaf total flavonoids fraction, Danshen total polyphenols fraction, Hawthorn leaf total flavonoids fraction.

### Animals

These procedures were approved by Science and Technological Committee and the Animal Use and Care Committee of TUTCM. Experiment was carried our in KK-A^y^ mice and C57BL/6 J (6 weeks old, equal numbers of male and female, weighing 18-22 g, Vital River Laboratory Animal Technology Co. Ltd., Beijing China) housed 2 to a cage and acclimated for 1 week before the experiments. All animals were allowed to eat a standard diet and drink *ad libitum*, and adapted to the experimental conditions at 22 ± 2°C, humidity 60 ± 5% with a fixed 12-h artificial light period. Administration protocol was as the same as our previous report [12]. Briefly, Test sample suspended in 5% acacia solution and vehicle (5% acacia solution) were given orally to KK-A^y^ mice once a day (16:00—17:00) and C57BL/6 J mice were administrated with the same volume of distilled water. The higher dosage was based on the typical clinical dose and fractions yield rate. Blood sample (*ca*. 0.2 ml) was collected from infraorbital venous plexus under ether anesthesia just before the experiment (0 d) and once every week after the administration. After 4 weeks administration, all the animals were fasted for 12 h. Soleus muscle, liver were collected and immediately frozen in liquid N_2_ and stored at -70°C until use for Western blot analysis.

### Western blot analysis

Frozen tissue was homogenized in ice-cold RIPA lysis buffer [150 mM NaCl, 0.5% Triton × 100, 50 mM Tris–HCl (pH 7.4), 25 mM NaF, 20 mM EGTA, 1 mM dithiothreitol (DTT), 1 mM Na_3_VO_4_, and 2 mM phenylmethyl sulfonyl fluoride (PMSF)] for 20 s on ice to give protein sample.

The protein concentration of the supernatant was measured by using the BCA protein assay kit (Yuanpinghao Bio Co. Ltd. China) with bovine serum albumin as standard. The insoluble protein solution was removed by centrifugation at 12000 rpm for 5 min. the supernatant was collected from the lysates and protein concentrations were determined using a Bio-Rad protein assay reagent (Bio-Rad Laboratories) following the manufacturer’s instructions. Equal amounts of proteins (40 *μ*g) were resolved by 8% SDS-polyacrylamide gel electrophoresis (SDS-PAGE) and transferred to polyvinylidene diflyoride membranes (Millipore, Bedford, MA). The normal proteins blots were blocked with 5% non-fat dry milk-TBST buffer [TBS containing 0.1% Tween-20] and phospho-proteins blots were blocked with commercial kits (Blocking One-p, Nacalai tesque Co. Ltd., Japan) for 1 h at room temperature. The membranes were incubated overnight at 4°C with 1:1000 dilution of antibodies for GYS1 (ab40810, Abcam Plc. UK (Ab)), *p*-GYS1 (ab81230, Ab), PI3K p85α ( #4292 s, Cell Signaling Technology Inc. MA, USA (CST)), *p*-PI3K p85α(#4228 s, CST), AKT (#9272 s, CST), *p*-AKT (#9271 s, CST), GluT4 (ab65267, Ab).

Equal lane loading was assessed using *β*-actin (SC-47778, Sigma Chemical Co., Santa Cruz, USA). The blots were rinsed seven times with TBST buffer for 3 min each. Washed blots were incubated with 1:10000 dilution of the horseradish peroxidase conjugated-secondary antibody (Zymed Laboratories, San Francisco, CA) for 1 h and washed five times with TBST buffer. The transferred proteins were visualized with an enhanced chemiluminescence detection kit (Millipore Co. Ltd. MA, USA). Blots were exposed to medical X-ray film (Fujifilm Europe, Germany) and quantified using a Universal Hood II and Quantity One imaging software (Bio-Rad Laboratories, Gladesville NSW, Australia). Results are expressed as a ratio of protein-to β-actin protein, normalised to the average control across all experiments.

### TG accumulation in HepG2 cell

The hepatic cell line HepG2 (IBMS, CAMS/PUMC, Beijing China) were maintained in Minimum Essential Medium (MEM) supplemented with 10% fetal bovine serum (FBS) and 1% penicillin-streptomycin under a humidified atmosphere of 5% CO_2_ in air. After growth to 80% confluence, cells were seeded at 4 × 10^4^ cells/mL on 48-well dish. After 24 hours incubation, the medium was switched to high glucose MEM and supplemented with 10% FBS and 0.2 mM oleic acid sodium salt, together with sample DMSO solution (final concentration of DMSO was less than 0.1%). After 48 hours incubation, the amount of intracellular triglycerides was determined with the Triglycerides kit (BioSino Bio-technology and Science Inc., China) after cell lysis.

### L-6 cell culture

L-6 rat skeletal muscle cells were (IBMS, CAMS/PUMC, Beijing China) incubated in α-MEM growth medium (37°C, 5% CO_2_) containing 100 U/ml penicillin, 100 μg/ml streptomycin and 10% FBS. Cells (2 × 10^4^ cells/ml) were differentiated into myotubes by changing culture media to α-MEM medium containing 2% FBS and maintained in culture for 1 week at 80% confluence. Before experiments, myotubes were incubated for 24 h at 37°C with α-MEM containing 2% FBS, 1 μM insulin, together with sample DMSO solution (final concentration was less than 0.1%). After maintained in DMEM medium supplemented with 25 mM glucose for 10 minutes, cells were washed three times with PBS.

### RNA extraction and cDNA synthesis

HepG2 and L-6 cells in 25 cm^2^ cell culture flasks (Corning, USA) were induced as previously described. Total RNA was isolated with TRIzol reagent (Invitrogen, USA). One microgram of RNA was reverse transcribed by the High Capacity cDNA Reverse Transcription Kit (Applied biosystems, USA) to obtain cDNA according to the protocols provided by the manufacturer. Briefly, the total reaction volume was 20 μl with the reaction incubated as follows in a PE-480 HYBAID (Perkin Elmer, USA): 10 min at 25°C, 120 min at 37°C, 5 min at 85°C, hold at 4°C.

### RT-PCR analysis

Synthesized cDNA was used in real-time RT-PCR (Bio-Rad Chromo 4 system) experiments using iQ SYBR Green Supermix and analyzed with Opticon Monitor software according to the manufacturer’s instructions.

Real-time PCR was performed with an Applied Biosystems 7500 Real-Time PCR System (Applied Biosystems, USA) using Power SYBR® Green PCR master mix (Applied Biosystems, USA) according to the protocols provided by the manufacturer. Briefly, PCR was performed in a final volume of 20 μl including 10 ng sample cDNA, 5 μM specific forward and reverse primers, and 10 μL Power SYBR® green PCR Master Mix. PCR reactions consisted of an initial denaturating cycle at 95°C for 10 min, followed by 40 amplification cycles: 15 s at 95°C and 1 min at 60°C. The primers were used as Tables 1 and 2. Results were presented as levels of expression relative to those of controls after normalization to GADPH using the 2^-ΔΔ*C*T^ method. Analysis was carried out in triplicates.

**Table 1:** Gene-specific primers used for quantitative real-time PCR of HepG2 cells

**Gene name**	**Forward**	**Reverse**
IRS-2	5′-TGGCAGTTCTCGCAGATGTT-3′	5′-GTCGACAGCCCTCCAATCAA-3′
AKT	5′-CCACGCTACTTCCTCCTCAA-3′	5′-TCCTCCTCCTCCTGCTTCTT-3′
GluT4	5′-CCGCTACCTCTACATCATCCA-3′	5′-GCTTCCGCTTCTCATCCTTC-3′
IRS-1	5′-TTCCGTAGTTCTGTAAGTCTGTCT-3′	5′-CCTCCAATATCATTCCACCTCCT-3′
GAPDH	5′-CCCATGTTCGTCATGGGTGT-3′	5′-TGGTCATGAGTCCTTCCACGATA-3′

**Table 2:** Gene-specific primers used for quantitative real-time PCR of L-6 cells

**Gene name**	**Forward**	**Reverse**
IRS-2	5′-AGGCTTGAAGCGGCTAAGTCTCAT-3′	5′-TGGCGCTTGGAATTGTGAGCAA-3′
AKT	5′-GTGTGGCAAGATGTGTATGAGAA-3′	5′-CAGGCGGTGTGATGGTGAT-3′
GluT4	5′-GGTTGGTGCCTATGTATGT-3′	5′-CGGATGATGTAGAGGTATCG-3′
IRS-1	5′-AATAGCCGTGGTGATTACAT-3′	5′-CAGAAGCAGAAGCAGAGG-3′
GAPDH	5′-AGACAGCCGCATCTTCTTGT-3′	5′-TGATGGCAACAATGTCCACT-3′

### Statistical analysis

Values are expressed as mean ± S.D. All the grouped data were statistically performed with SPSS 11.0. Significant differences between means were evaluated by one-way analysis of variance (ANOVA) and Tukey’s Studentized range test was used for post hoc evaluations. *P* < 0.05 was considered to indicate statistical significance.

## Results

### Protein expression analysis of muscle and liver in KK-A^y^ mice

**Figure 1:**
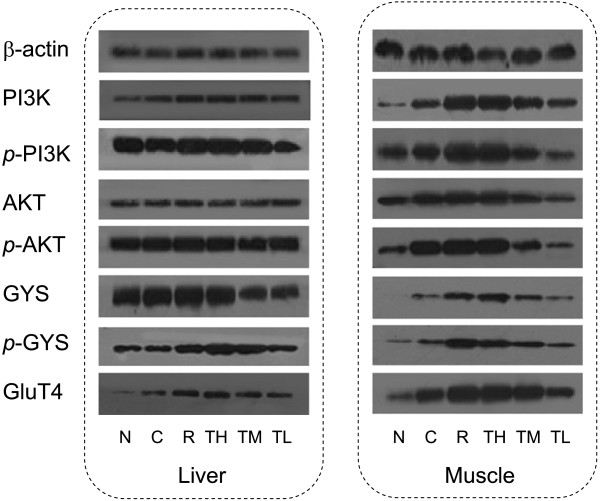
Western blot analysis of liver and soleus muscle tissue in KK-Ay mice.

**Figure 2:**
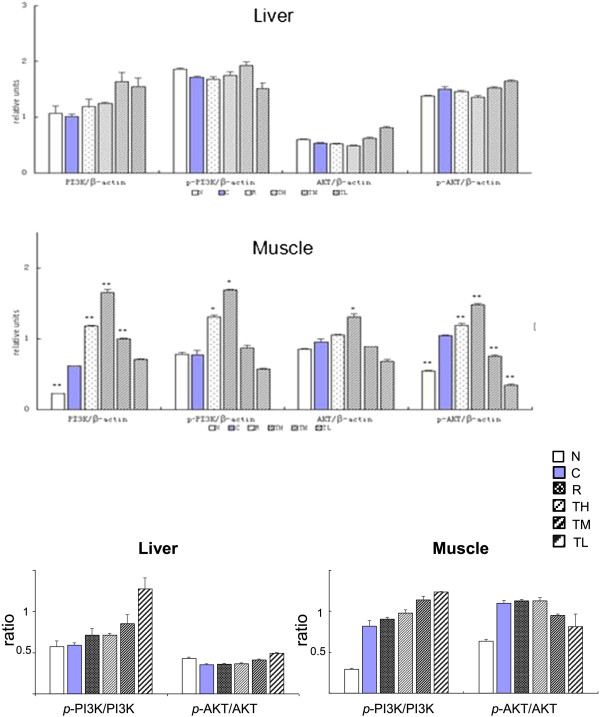
Western blot analysis of liver and soleus muscle tissue in KK-Ay mice.

To evaluate the effect of TZQ-F on PI3K signaling pathway *in vivo*, proteins expression were analyzed in liver and muscle samples of KK-A^y^ mice after 4 weeks administration (Figure 1). In liver, total PI3K in controls group slightly increased in TZQ-F mice, while no detectable PI3K, AKT and their phosphorylation type changes in Rosiglitazone (R, 10 mg/kg/day, *p.o*.) and TZQ-F (100 (TL), 200 (TM) and 500 (TH) mg/kg/day, *p.o*.) groups. On the contrary, phosphorylated GYS and GluT4 were up-regulated after Rosiglitazone and TH treatments. In muscle, up-regulation of PI3K, AKT, GYS and their phosphorylation type, as well as GluT4, was deteced in Rosiglitazone and TH treated mice in a dose-dependent manner. Both in muscle and liver, PI3K was slightly, but no significantly phosphorylated by TZQ-F administration. Ratio of phosphorylated AKT and total AKT showed no significant changes between TZQ-F groups and control group. (Figure 2).

### TZQ-F inhibited free fatty acid induced triglyceride accumulation in HepG2 cells

**Figure 3:**
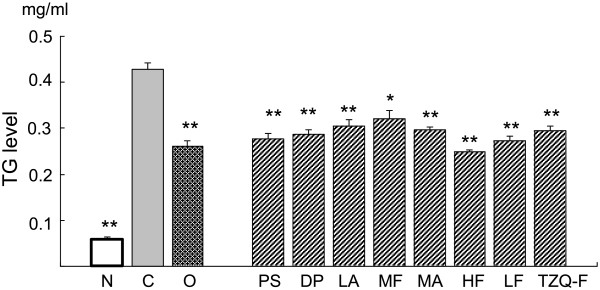
Effect of TZQ-F and its fractions on TG accumulation in HepG2 cell.

In our previous study, we investigated TZQ-F and fractions with the range of 0.1 to 10 μg/ml. The results showed that each fraction has a moderate effect in the regulation of lipid metabolism at a concentration of 1 μg/ml, This concentration (1 μg/ml) was suitable to explore the contribution of each fraction to the target tissues. HepG2 cells were treated with 1 μg/ml TZQ-F and its fractions. Under the concentrations, no detectable treatment-related changes were observed in cell viability (data not shown). Adding 0.2 mM oleic acid, many lipid droplets were observed in controls, which indicated the successful induction of triglyceride accumulation. As shown in Figure 3, compared to the untreated cells, TZQ-F and its fractions significantly suppressed the accumulation of triglyceride in HepG2 cells induced by free fatty acid.

### Gene expression and protein expression analysis in HepG2 cells

**Figure 4:**
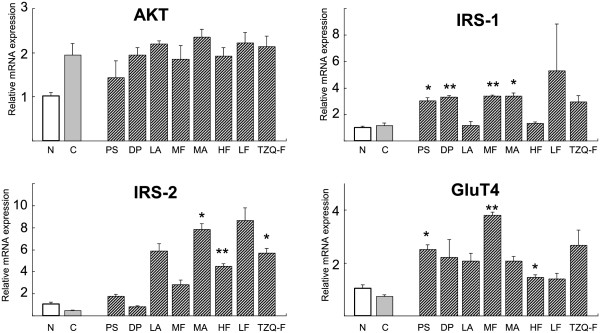
Effect of Effect of TZQ-F and its fractions on AKT, IRS-1/2 and GluT4 gene expression in HepG2 cell.

**Figure 5:**
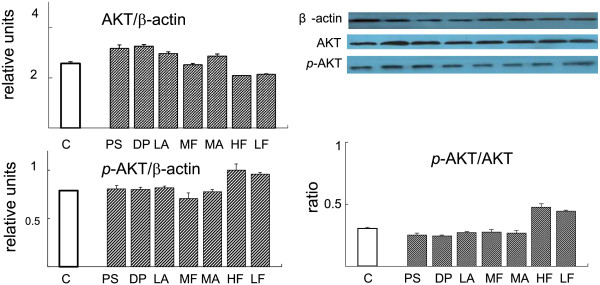
Effect of TZQ-F fractions on AKT proteins expression in HepG2 cell.

According to the result *in vivo*, AKT, IRS-1/2 and GluT4 gene expression was analyzed in HepG2 cells. As shown in Figure 4, TZQ-F showed no effects on AKT, IRS-1 and GluT4 expression, while IRS-2 were up-regulated by 10 folds. On the other hand, Red Paeony total saponins fraction, Danshen total polyphenols fraction, Mulberry leaf total flavonoids fraction and alkaloids fraction showed up-regulation effects on IRS-1 expression. Mulberry leaf total alkaloids fraction and Hawthorn leaf total flavonoids fraction showed up-regulation effects on IRS-2 expression. Red Paeony total saponins fraction, Mulberry leaf total flavonoids fraction and Hawthorn leaf total flavonoids fraction showed up-regulation effects on GluT4 expression.Protein expression analysis also shows that AKT, *p*-AKT dose not change (Figure 5).

### Gene expression and protein expression analysis in L-6 cells

**Figure 6:**
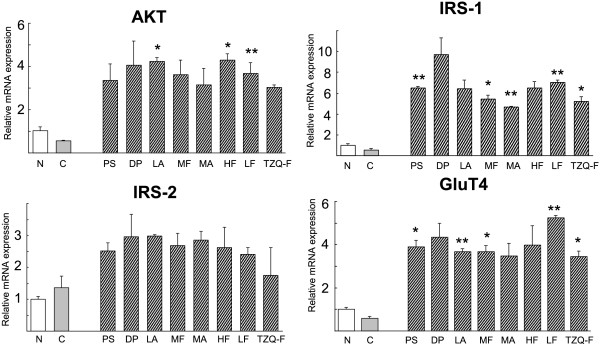
Effect of TZQ-F and its fractions on AKT, IRS-1/2 and GluT4 gene expression in L-6 myotube.

After exposure in 25 mM glucose and 1 μM insulin for 10 minutes, GluT4 in L-6 myotube cell was 42% reduced compared to the normal group (treated with 25 mM glucose for 10 minutes) at mRNA level. TZQ-F and its fractions (1 μg/ml, respectively) enhanced GluT4 by approximately 6 to 8 folds (Figure 6). Among them, the up-regulation by Lotus leaf total flavonoids fraction was the most notable. TZQ-F led to 5 folds increasing on GluT4.

**Figure 7:**
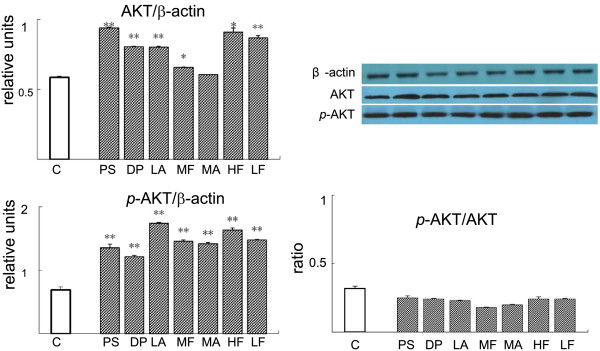
Effect of TZQ-F fractions on AKT proteins expression in L-6 myotube.

TZQ-F and its fractions treatment also increased IRS-1 and AKT in L-6 myotube cells (Figure 6). Red Paeony total saponins fraction, Mulberry leaf total flavonoids fraction and Mulberry leaf total alkaloids fraction showed statistically up-regulation effects on IRS-1 expression by approximately 10 to 20 times. Hawthorn leaf total flavonoids fraction, Lotus leaf total alkaloids fraction and Lotus leaf total flavonoids fraction showed significantly up-regulation effects on IRS-1 expression. On the contrary, no significance of IRS-2 was detected. TZQ-F and its fractions (1 μg/ml, respectively) have been shown to activated AKT at protein levels. Compared to the control group, TZQ-F and its fractions (1 μg/ml, respectively) enhanced *p*-AKT by approximately 3 folds. Among them, the up-regulation by Lotus leaf total alkaloids fraction was the most notable (Figure 7).

## Discussion

In the previous report, we found that TZQ-F showed decreasing effects on serum total cholesterol, triglyceride, glucose, and cholesterol levels in high-fat diet induced rat model [11]. The mechanism was partly clarified on AMPK signaling pathway [12].In this paper, we further demonstrated TZQ-F played its role on glucose and lipid regulation by activation of PI3K, AKT and GSK-3β in skeletal muscle.

In clinical traditional Chinese medicine, TZQ-F was widely used for pre-diabetes patient, whose serum glucose was in the range from 100 to 125 mg/dL, with a slight abnormal blood glucose level after the fasting plasma glucose test. [13] In this period, glucose uptake rate under insulin stimulation is thought to be decreased as an earliest event in diabetes trajectory. By contrast, insulin resistance in liver is a very late event in developing hyperglycemia [14]. Activation of AKT was an effective way to diminish development of pre-diabetes to diabetes [15].

PI3K signaling pathway plays an important role in insulin resistance, including insulin activation, GluT translocation and glycogen synthesis. Rosiglitazone, one of thiazolidinedione drug used for the treatment of type 2 diabetes, has a potential effect on insulin resistance through PI3K signaling pathway [16,17] to regulate expression and activation of IRS 1/2, GluT4 , PPAR-γ, PI3K and AKT to increases insulin stimulated glucose transport [18].

AKT, also known as protein kinase B (PKB), a serine/ threonine kinase, is a central player in processes downstream of activated PI3K signaling, involving in the regulation of both glycolytic and oxidative metabolism [19]. AKT activation showed tissue-specific differences between insulin-sensitive target tissues including liver, skeletal muscle and adipose tissue [20]. AKT activation is dependent on IRS-1-dependent PI3K in muscle, but dependent on both IRS-1 and 2- dependent PI3K in liver [21]. Accordingly, a lot of literatures reported that Thiazolidinedione can improve AKT activation in rodent diabetes models’ muscle, but do not alter it in liver [22]. The experimental results agree well with above AKT activation mechanism. In L-6 myotube, fractions of TZQ-F showed up-regulation effects on AKT gene expression, especially Lotus leaf total alkaloids, Hawthorn leaf total flavonoids and Lotus leaf total flavonoids fraction. In liver, insulin receptors were selectively activated at IRS-2 by TZQ-F, but not IRS-1, which indicated that glucose homeostasis effect of TZQ-F is not related with PI3K/AKT signaling pathway. However, in muscle, insulin receptors were selectively activated at IRS-1 by TZQ-F, but not IRS-2. The results were further confirmed by gene expression analysis in HepG2 and L-6 cell.

AKT activity increase the translocation of GluT4 to the plasma membrane in muscle and fat cell, thus increasing the uptake of glucose into cells [23]. Several research groups reported that GluT4 content in skeletal muscle is highly related to insulin resistance, primary defect in insulin-stimulated muscle glucose uptake can cause insulin resistance in other insulin-responsive organs, such as liver and adipocytes, and subsequently result in the development of diabetes [24]. In this study, TZQ-F treated groups significantly increased GluT4 in skeletal muscle, which indicated that TZQ-F may play a dominant role in glucose homeostasis. TZQ-F showed a tendency toward increasing GluT4 gene level in KK-A^y^ mice muscle compared with control group, which was also confirmed in L-6 myotube cells. Further studies are required to investigate whether TZQ-F affect the translocation of GluT4 in cellular level, and other IRS kinases, such as S6K1 and PKC.

## Conclusion

As a conclusion, we partly revealed pre-diabetes prevention mechanism of TZQ-F, which at least, related to increasing effect on IRS-1-dependent PI3K/AKT signaling pathway in muscle.

## Competing interests

The authors declare that they have no competing interests.

## Authors’ contributions

JG, JL, YA, XL, QQ and YW performed the experiments under the supervision of YZ and TW, YZ and TW wrote the manuscript draft, which was read and edited by all authors. All authors read and approved the final version of the manuscript.

## Pre-publication history

The pre-publication history for this paper can be accessed here:

http://www.biomedcentral.com/1472-6882/14/198/prepub
